# Preconception Interventions in Women at High Risk of Developing Gestational Diabetes: A Systematic Review

**DOI:** 10.1007/s10995-026-04236-5

**Published:** 2026-02-19

**Authors:** Catherine V. George, Dominika Bhatia, Olivia Righton, Zeinab El Dirani, Sara L. White, Lucilla Poston, Ola Quotah, Danielle A. J. M. Schoenaker, Fiona Lavelle, Claire M. Timon, Angela C. Flynn, Pauline Dunne

**Affiliations:** 1 School of Population Health, RCSI University of Medicine and Health Sciences, Dublin, Ireland; 2 Department of Nutritional Sciences, School of Life Course and Population Sciences, King’s College London, London, UK; 3 Department of Women and Children’s Health, School of Life Course and Population Sciences, King’s College London, London, UK; 4 Department of Diabetes and Endocrinology, Guy’s and St Thomas’ Hospital NHS Foundation Trust, London, UK; 5 School of Human Development and Health, Faculty of Medicine, University of Southampton, Southampton, UK; 6 MRC Lifecourse Epidemiology Centre, University of Southampton, Southampton, UK; 7 NIHR Southampton Biomedical Research Centre, University of Southampton, University Hospital Southampton NHS Foundation Trust, Southampton, UK

**Keywords:** Gestational diabetes, Intervention, Preconception, Diet, Physical activity, Randomised controlled trials, Systematic review

## Abstract

**Introduction:**

Gestational diabetes mellitus (GDM) is a common pregnancy-related complication. The modest benefits of interventions to prevent GDM in women with high risk during pregnancy has shifted the focus to the preconception period. However, research on the effectiveness of preconception interventions in women who are more likely to develop GDM is lacking. This review aimed to assess the effect of preconception interventions, including behavioural strategies, supplementation, and pharmacological treatments on reducing the incidence of GDM.

**Methods:**

MEDLINE, EMBASE and the Cochrane Central Register of Controlled Trials were first searched in February 2023 and updated in December 2025 (PROSPERO CRD42020177976). Randomised controlled trials (RCTs) evaluating dietary/physical activity (PA)/combined, nutritional supplementation or pharmacological intervention in the pre-pregnancy period in women at high risk of developing GDM conducted in any country and reported in English were included. The pre-pregnancy period was defined as the period before and/or between pregnancies. A narrative synthesis was conducted in line with the Synthesis Without Meta-analysis guidelines, and each study was assessed using the Cochrane risk of bias tool.

**Results:**

Six RCTs, equating to nine publications (*n* = 909 participants in total) were included. Two interventions were conducted during the pre-pregnancy period and discontinued upon conception, and the remaining four were delivered pre-pregnancy and continued throughout pregnancy. Five trials focused on modifying both dietary intake and PA and one trial modified diet only. No studies reported a significant effect of preconception behaviour change intervention on GDM development; however, five of the trials were underpowered to do so.

**Discussion:**

Limited evidence fitted this review’s inclusion criteria, highlighting a considerable research gap. Future well designed, adequately powered RCTs of behaviour change and/or pharmacotherapy in women at higher risk for developing GDM are necessary to inform preconception care guidelines to improve the immediate and long-term health of women and their infants.

**Supplementary Information:**

The online version contains supplementary material available at 10.1007/s10995-026-04236-5.

## Significance

What is already known on this subject?

What does this study add

The modest benefits of intervention to prevent GDM during pregnancy in women at higher risk has shifted focus to the preconception period, highlighting the need for early intervention to reduce pregnancy complications and long-term maternal and child health risks. Additionally, preconception health is suboptimal among women at increased risk of GDM, including those living with overweight or obesity or with a history of GDM.

This review evaluated interventions to reduce GDM which were initiated in the preconception period and highlights a critical evidence gap. Only six RCTs were identified and while the findings suggest that preconception diet and physical activity intervention shows some benefits, impact on GDM prevention remains uncertain due to underpowered studies. This review highlights the urgent need for adequately powered RCTs that specifically target the preconception period in women likely to develop GDM. Research gaps on pharmacological interventions to address preconception obesity were also identified in this review.

**Supplementary Information:**

The online version contains supplementary material available at 10.1007/s10995-026-04236-5.

Gestational diabetes mellitus (GDM) is a metabolic disorder defined as hyperglycaemia first recognised during pregnancy that resolves after birth (McIntyre et al., 2019). GDM is one of the most common pregnancy-related complications, affecting approximately 14% of pregnancies worldwide, with prevalence varying across countries, populations, screening strategies and diagnostic criteria (Wang et al., 2022). While consensus is lacking on optimal screening strategies, timing of and/or diagnostic criteria for GDM, prevalence continues to rise globally due to increases in maternal age and obesity, and associated cardiometabolic risk factors (Eades et al., 2017; Lavery et al., 2017; Muche et al., 2019; Tutino et al., 2014). Women with GDM have higher risks of developing gestational hypertension and pre-eclampsia, delivery complications such as caesarean delivery, and future risk of recurrent GDM, type 2 diabetes (T2D) and cardiovascular disease (Metzger et al., 2009; Ye et al., 2022). GDM in multiple pregnancies further exacerbates this future chronic disease risk (Mussa et al., 2024). Furthermore, infants of women with GDM are at risk of macrosomia, being born large for gestational age, pre-term delivery, and developing metabolic disease in later life (Metzger et al., 2009; Ye et al., 2022).

Individual level risk factors for GDM are generally classified as non-modifiable, (i.e. age, family history of diabetes, previous GDM, polycystic ovarian syndrome) or modifiable (i.e. overweight, obesity, physical activity (PA), diet) (Sweeting et al., 2024). Efforts to prevent GDM during pregnancy have mainly focused on reducing insulin demand and targeting modifiable risk factors associated with adverse maternal and fetal outcomes (Simmons et al., 2024; Sweeting et al., 2024). In a systematic review of diet and PA interventions to reduce gestational weight gain (GWG), after studies with methodological limitations were excluded, there was no evidence of an intervention effect on clinical outcomes, including GDM (Dodd et al., 2024a). More recently, diet and PA interventions during pregnancy were shown to prevent GDM, with effects varying according to diagnostic criteria(Allotey et al., 2026). However, the effect of antenatal intervention in women at higher risk is less clear. Previous evidence from a systematic review showed only modest benefit of behaviour change (e.g. diet and PA) to prevent GDM in women identified at higher risk of developing the condition (Quotah et al., 2024).

Suboptimal success in preventing GDM in those at higher risk during pregnancy has directed focus towards the preconception period. The 2018 Lancet series on preconception health highlighted the critical need to expand interventions to the period before conception to reduce the risk of pregnancy complications and associated longer-term risks to the woman and her offspring, rather than waiting to intervene during pregnancy (Barker et al., 2018; Fleming et al., 2018; Stephenson et al., 2018). This is particularly important as preconception health has been shown to be suboptimal in the general population of women (Stephenson et al., 2021) and in women with previous GDM, where high levels of obesity have been reported. Survey data from a digital pregnancy planning tool found that almost half of women with previous GDM enter pregnancy with obesity, compared to a quarter of women without any history of diabetes (Flynn et al., 2023).

Although observational evidence suggests that preconception weight loss, improved diet quality and increased PA levels are associated with reduced GDM risk (Sampathkumar et al., 2023; Schoenaker et al., 2020), there is a paucity of evidence from randomised controlled trials (RCTs). Preconception diet and PA interventions were not associated with a significant difference in rates of GDM in a recent systematic review (Poprzeczny et al., 2025), however, additional research is needed in women at higher risk of developing GDM, to inform the development of guidelines to improve preconception health of women with risk factors for developing the condition. This systematic review, therefore, aimed to assess the effect of preconception interventions, including behaviour change, nutritional supplementation, and pharmacological treatments, on GDM risk in women at higher risk of developing the condition.

## Methods

This systematic review was conducted in accordance with the Synthesis without meta-analysis (SWiM) guidelines (See Online Resource 1) (Campbell et al., 2020) and was reported in alignment with the Preferred Reporting Items for Systematic Reviews and Meta-Analyses (PRISMA) recommendations (See Online Resource 2) (Moher et al., 2009).

### Search Strategy

The first step of this review involved updating a prior literature search, which aimed to assess the effect of preconception and pregnancy interventions in women at high risk of developing GDM, to identify new published trials (PROSPERO CRD42020177976). The details of the previous review have been described in another publication (Quotah et al., 2024). In brief, MEDLINE, EMBASE and the Cochrane Central Register of Controlled Trials were searched, initially in February 2023, updated in February 2025 and repeated in December 2025 (See Online Resource 3). Backward and forward citation searching of was undertaken to supplement the database searches.

### Eligibility Criteria

Inclusion and exclusion criteria has been previously described (Quotah et al., 2024). In brief, to be eligible for inclusion, studies had to meet the following criteria: (i) randomised controlled trials (RCTs) that evaluated any intervention in the pre-pregnancy period or during pregnancy compared with no intervention, another type of intervention, placebo or standard care; (ii) women identified as higher risk of developing GDM, using any risk stratification in the preconception period or in early pregnancy; (iii) data reporting GDM as a primary or secondary pregnancy outcome (iv) conducted either low- and middle-income or high-income countries. All diagnostic criteria for GDM were deemed acceptable. Studies meeting the following criteria were excluded: (i) non-randomised, observational, or case studies; (ii) abstracts, reviews, letters, commentaries and editorials; (iii) women aged less than 18 years or older than 50 years; (iv) studies designed to treat GDM; (v) interventions starting too late in pregnancy (> 28 weeks’ gestation) (vi) studies not reported in English. As the focus of this review was on preconception interventions only, any RCTs which targeted pregnancy, and which did not initiate an intervention in the pre-pregnancy period were excluded. Preconception was defined as the period before or between pregnancies.

### Study Selection

All studies identified in the search process were imported into EndNote reference management software to eliminate duplicate publications and then into Rayyan screening management software (Ouzzani et al., 2016). Citations were screened in duplicate by independent reviewers (CVG, DB, OR, ZED) in two phases: (i) titles and abstracts and (ii) full-text articles. Disagreements were resolved by consensus with a third reviewer or group discussion.

### Data Extraction

Data extraction was carried out in duplicate by the reviewers (CVG, DB, OR) independently using a standardised table created for this review. Items for data extraction included title, authors, publication year, study design, country, aim, population characteristics, inclusion and exclusion criteria, period of intervention (preconception and/or preconception and pregnancy), intervention characteristics including description of intervention (including type, timing, duration, delivery method), feasibility and acceptability of intervention methods, behavioural change theory framework and adverse pregnancy outcomes. Any disagreement between reviewers was resolved through a consensus opinion.

### Data Synthesis

Due to the limited studies, heterogeneity of study design, interventions and outcomes, conducting a meta-analysis was not appropriate (See Online Resource 1). Studies were divided into pre-pregnancy only or combined pre-pregnancy and pregnancy periods. The data are presented in textual and tabular formats, offering a comprehensive summary of the findings.

### Risk of Bias Assessment

The Cochrane risk of bias tool (Sterne et al., 2019) was used to assess the bias of each study included according to the Cochrane Handbook for Systematic Reviews of Interventions, version 6.3 (Higgins et al., 2024). The domains assessed included randomisation method, allocation concealment, incomplete outcome data, selective outcome reporting and other potential sources of bias. Any disagreement between reviewers (CVG, DB, OR) was resolved through a consensus with a fourth reviewer (ACF). The overall risk was determined, and studies were classified as ‘low risk of bias’, ‘some concerns’ or ‘high risk of bias’. The robvis tool was used to build the risk-of bias plot (McGuinness & Higgins, 2020).

## Results

**Fig. 1:**
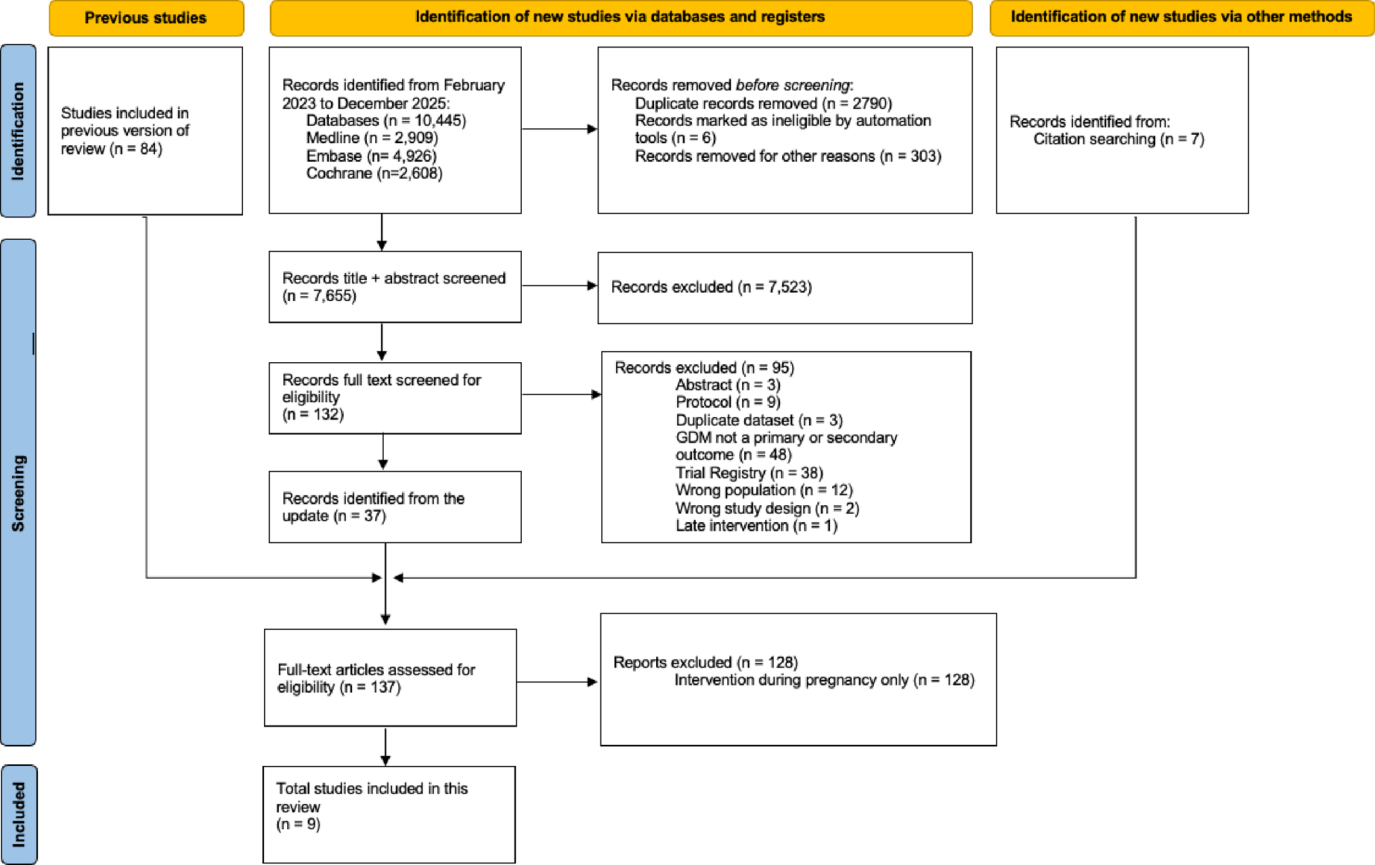
PRISMA flow chart

Six RCTs (Bogaerts et al., 2025; LeBlanc et al., 2021b; Phelan et al., 2023; Price et al., 2021; Rönö et al., 2018; Sujan et al., 2025), equating to nine publications (LeBlanc et al., 2021a, 2022; Valkama et al., 2018), were included in this review (see Fig. 1), two of which were identified previously (Quotah et al., 2024). The majority of studies were excluded because the intervention occurred entirely during pregnancy without a pre-pregnancy component, studies were ongoing RCTs (without published results), or studies did not include GDM as a primary or secondary outcome. Intervention details for all six trials were obtained from previously published protocols (Bogaerts et al., 2017; LeBlanc et al., 2016; Phelan et al., 2021; Price et al., 2018; Rönö et al., 2014; Sujan et al., 2023) and other reports (Bogaerts et al., 2020).

### Characteristics of Included RCTs

All six trials were conducted in high-income countries: Australia (Price et al., 2021), Belgium (Bogaerts et al., 2025), Finland (Rönö et al., 2018), Norway (Sujan et al., 2025), and the United States of America (USA) (LeBlanc et al., 2021b; Phelan et al., 2023) (Table 1). Three trials aimed to determine the effect of pre-pregnancy weight loss on GDM occurrence, GWG and other pregnancy outcomes (LeBlanc et al., 2021b; Phelan et al., 2023; Price et al., 2021). Two trials aimed to evaluate the effect of a pre-pregnancy dietary and PA behaviour change intervention on glucose tolerance (Sujan et al., 2025) and GDM diagnosis (Rönö et al., 2018), respectively. One trial aimed to assess the effect of behaviour change intervention in women with a history of excessive GWG (Bogaerts et al., 2025). To define a population at high risk, studies differed in the preconception GDM risk factors they considered. Five trials recruited women living with overweight or obesity (ranging from ≥ 25 to “between 30 and 50” kg/m^2^) (LeBlanc et al., 2021b; Phelan et al., 2023; Price et al., 2021; Rönö et al., 2018; Sujan et al., 2025) and/or a history of GDM (Phelan et al., 2023; Rönö et al., 2018; Sujan et al., 2025). One trial recruited women with a history of excessive GWG in previous pregnancy (Bogaerts et al., 2025). All trials recruited women planning a pregnancy within the next 6 months to 3 years. The total sample size was 909 participants with primary or secondary outcome GDM data, which ranged across studies from 57 to 390 participants. Studies included women before a first pregnancy and/or next pregnancy.

**Table 1:** Characteristics of studies

References	Country	Study design	Study aim	Study timepoint	Inclusion criteria	Study population at randomisation	Study group at analysis	GDM diagnostic criteria
Bogaerts et al. (2025)	Belgium	Prospective Multicenter-centre RCT	To assess the effectiveness of a comprehensive lifestyle intervention that commenced in the interpregnancy and continued through the subsequent pregnancy in women with a history of excessive GWG	Pre-Pregnancy & Pregnancy	Excessive GWG beyond the National Academy of Medicine recommendations; age ≥ 18 years; proficient in Dutch; capable of using a smartphone	*N* = 1,**450** **Intervention** *n* = 724 **Control** *n* = 726	*N* = 390 Age: 30 ± 3.4 years, GDM in previous pregnancy: 12 (3.1%) **Intervention** *N* = 193 Age: 30 ± 3.3 years; GDM in previous pregnancy: 6 (3.1%) **Control** *N* = 197 Age: 30 ± 3.5 years, GDM in previous pregnancy: 6 (3.1%)	Flemish guidelines: Universal early screening (before 20 weeks) for overt diabetes, with elevated FPG indicating early GDM. Otherwise, IADPSG with 75 g OGTT done 24–28 weeks
LeBlanc et al. (2021a, 2021b)	USA	Single-centre RCT	To determine whether pre-pregnancy weight loss reduces gestational weight gain and improves pregnancy outcomes.	Pre-Pregnancy & Pregnancy	BMI ≥ 27 kg/m^2^; ≥ 18 to 40 years old; planning a pregnancy in the next 2 years	*N* = 326 BMI: 36.8 (7.3) kg/m² Age: 31.7 (3.8) years **Intervention** *n* = 164 **Control** *n* = 162	*N = 169 (51.8%)* BMI at study entry: 34.8 (5.8) kg/m² Age: 31.3 (3.5) years **Intervention** *n* = 89 (51%) BMI at study entry: 34.9 (6.0) kg/m² Age: 31.6 (3.5) years **Control** *n* = 80 (49%) BMI at study entry: 30.9 (3.5) kg/m² Age: 34.7 (5.5) years	ADA At first prenatal visit, participants randomly assigned to either a 1-step or 2-step screening process, which remained consistent throughout pregnancy.
Phelan et al. (2023)	USA	2-site, parallel-group, RCT	To determine the effects of a pre-pregnancy weight loss intervention on gestational diabetes mellitus recurrence in women with overweight/obesity and previous GDM.	Pre-Pregnancy	Previous GDM; BMI ≥ 25 kg/m^2^; ≥ 18 years; planning a pregnancy within the next 1 to 3 years	*N* = 199 **Intervention** *n* = 105 **Control** *n* = 94	**N = 63 (33%)** BMI at study entry: 32.9 (6.5) kg/m² Age: 32.7 (4.5) years Years since previous GDM: 1.7 (1.5) **Intervention** *n* = 38 (37%) BMI at study entry: 33.4 (6.7) kg/m² Age: 32.4 (4.2) years Years since previous GDM 1.9 (2.6) **Control** *n* = 25 (28%) BMI at study entry: 32.1 (5.8) kg/m² Age: 32.9 (5.1) years Years since previous GDM 1.4 (1.2)	ADA Carpenter and Coustan criteria and 2 abnormal values on a study-measured 3-hour OGTT
Price et al. (2021)	Australia	Two-arm, parallel-group RCT	To determine if nonsurgical, substantial weight loss can be achieved in women with obesity prior to pregnancy using a modified VLED program and to determine whether this weight loss improves glucose metabolism and obesity-related adverse pregnancy outcomes for both women and offspring.	Pre-Pregnancy & Pregnancy	BMI between 30 and 55 kg/m^2^; planning a pregnancy within the next 6 to 12 months	*N* = 164 **Intervention** *n* = 85 BMI at study entry: 39.5 (0.7) kg/m² Age: 32.1 (5.2) years **Control** *n* = 79 BMI at study entry: 37.9 (0.7) kg/m²; Age: 32.6 (3.6) years	**N = 57 (34.7%)** **Intervention** *n* = 35 (41%) **Control** *n* = 22 (28%)	IADPSG 75 g 2-hour OGTT at 26 to 28 weeks as part of standard maternity care in Australia
Rönö et al. (2018)	Finland	Multi-centre RCT	To evaluate the efficacy of a combined diet and physical activity intervention during pre-pregnancy in GDM prevention.	Pre-Pregnancy & Pregnancy	Previous GDM or BMI ≥ 30 kg/m^2^; ≥ 18 years old; planning a pregnancy within 1 year	*N* = 228 **Intervention** *n* = 116 BMI at study entry: 30.4 ± 6.1 kg/m² Age: 33 (4) years Previous GDM: *N* = 94 (81%) **Control** *n* = 112 BMI at study entry: 29.4 ± 5.9 kg/m² Age: 32 (4) years Previous GDM *N* = 88 (79%)	**N = 128 (56.1%)** **Intervention** *n* = 65 (56%) BMI at study entry: 30.5 (6.3) kg/m²; Age: 32 (5) years; Previous GDM *n* = 51 (78%) **Control** *n* = 63 (56%) BMI at study entry: 28.1 (5.7) kg/m²; Age: 32 (4) years; Previous GDM *n* = 51 (78%)	ADA 75 g 2-hour OGTT at 12–16 weeks of gestation, and if normal, it was repeated at 24–28 weeks of gestation.
Sujan et al. (2025)	Norway	Single-centre RCT	To determine the effect of a pre-pregnancy lifestyle intervention on glucose tolerance in people at higher risk of GDM.	Pre-Pregnancy & Pregnancy	At least one of the following criteria: BMI ≥ 25 < 40 kg/m2, GDM in a previous pregnancy, fasting plasma glucose > 5.3 mmol/L, previous newborn > 4.5 kg, or Non European ethnicity; contemplating pregnancy within the next 6 months	*N* = 167 **Intervention** *n* = 84 Age: 30.2 ± 3.1 years; BMI: 29.2 ± 4.9 kg/m² **Control** *n* = 83 Maternal age: 30.3 ± 3.2 years; BMI: 29.1 ± 4.5 kg/m²	**N = 164 (98.2%)** were included in ITT analysis of secondary outcomes. **Intervention** *n* = 83 **Control** *n* = 83 **N = 102 (61.1%)** became pregnant and were included in analysis of primary outcome. **Intervention** *n* = 49 **Control** *n* = 53	WHO 2013 Fasting plasma glucose 5.1–6.9 mmol/L or two hour plasma glucose 8.5–11.0 mmol/L, or both, after a 75 g glucose load. Measured at gestational weeks 12 and 28.

_*ADA* American Diabetes Association, *BMI* Body Mass Index, *GDM* Gestational Diabetes Mellitus, *GWG* Gestational Weight Gain, *IADPSG* International Association of Diabetes and Pregnancy Study Group, *OGTT* Oral Glucose Tolerance Test, *RCT* Randomised Controlled Trial, *VLED* Very−Low Energy Diet, *WHO* World Health Organization_

### Pre-pregnancy only Interventions

Two interventions were conducted during the pre-pregnancy period and discontinued upon conception. Phelan et al. (2023) conducted a 2-site parallel-group RCT in the USA, which aimed to determine the effects of a pre-pregnancy weight loss intervention on GDM recurrence in women with overweight/obesity and previous GDM. At study entry, both groups (intervention, *n* = 105; control, *n* = 94) received standard care and education (2 × 20 min meetings). The intervention group also received a diet, PA, behavioural health, and weight loss intervention. In the first 16 weeks, participants aimed to achieve 10% weight loss and then maintain weight loss until conception. The intervention duration was 49.8 ± 40 weeks (Table 2). There was a significant difference in mean preconception weight loss during these 16-weeks between the intervention and control group (intervention, *n* = 34, 4.8 (range: 3.5–6.0) kg (5.7%); control, *n* = 22, 0.7 (range: − 0.9 to 2.3) kg (0.7%); *P* = 0.001). However, the intervention had no effect on the primary outcome of GDM recurrence rate relative to the control or secondary outcome of GWG between groups. The intervention improved reported weight control practices and cognitive restraint over the first 16 weeks. Independent of group allocation, increasing weight loss between study entry and last weight measured before pregnancy predicted 21% lower odds of GDM recurrence (OR, 0.79; *P* = 0.008). The study also suggested that weight loss ≥ 5% before conception reduced the odds of GDM recurrence by 82% (OR, 0.18 [0.04–0.88]; *P* = 0.03) (Table 3).

**Table 2:** Characteristics of interventions

References	intervention type	Study groups	Intervention delivery & timing	Intervention content	Intervention measurement tools & assessment	Behavioural recommendations	Behaviour Change Theory underpinning	Acceptability and Feasibility measurement tools
Bogaerts et al. (2025)	Combined lifestyle and behaviour-change intervention with in-person coaching with a digital (eHealth) app	**Intervention** **Phase 1**: After childbirth before subsequent pregnancy. One-on-one coaching sessions with a trained health professionals, including nutritionists, psychologists, midwives, and/or nurses. Focused on diet, PA and mental wellbeing. Each participant was assigned to a specific coach. Access to the INTER-ACT application an E-Health application	**Phase 1**: 4 face-to-face coaching sessions 6, 8, 12 weeks, and 6 months	**Phase 1**: Coaching sessions focused on dietary habits, eating behaviours, food intake, physical activity and mental wellbeing stressors and the importance of social support. Emphasis on weight management and participants returning to their preconception weight.	INTER-ACT application, included an activity tracker, weighing scale and self-monitoring applications for weight, PA, mental wellbeing and stress levels, bolstering coaching impact, motivation, and study adherence.	INTER-ACT application promoted healthy behaviours, including nutrition, PA, and mental wellbeing.	Motivational interviewing techniques such as the Ask-Tell-Ask method, supported by the Prochaska and DiClemente’s transtheoretical model. Behavioural change techniques including goal setting, action planning, individual tips and tricks for improving lifestyle, self-monitoring, and encouragement.	Coaching session attendance and app usage.
		**Phase 2**: During subsequent pregnancy.One-on-one coaching sessions and continued access of the INTER-ACT application. **Control**:Received home visits at 6 weeks and 6 months postpartum, and subsequent every 6 months until next pregnancy	**Phase 2**: 3 sessions: before 15 weeks, week 20, and week 32 of gestation in subsequent pregnancy	**Phase 2**: Coaching sessions focused on nutrition and dietary support for pregnancy and GWG, PA and discussions on sleep during pregnancy. Emphasis on achieving healthy GWG				
LeBlanc et al. (2021a, 2021b)	Combined lifestyle intervention (diet + PA)	**Intervention**: Individualised 20- to 30-minute telephone counselling sessions with the health coach, a trained behavioural interventions, and access to a personalised intervention website **Control**: Routine prenatal care + information on having a healthy pregnancy (5–10 min)	6 weekly sessions over 6 months, then monthly sessions for up to 18 months or until end of pregnancy	**Diet**: Calorie control to be within customised target: Follow the meal plan, control portions, limit sweets and sugar-sweetened beverages. - **DASH diet**: 8–12 servings of fruits/vegetables, 3 servings of low-fat dairy, limit unhealthy fats, aim for 6 small meals and snacks. **PA**: 10,000 steps/day, work up to 60 min of moderate intensity exercise, follow doctor’s advice	**Measurement timepoints**: Baseline (preconception) and 20 weeks’ gestation. **Diet**: 24-hour recall with two target recalls at two timepoints. Recalls were collected remotely using either the 2014 or 2016 version of the Automated Self-Administered 24‐h (ASA24^®^) Dietary Assessment Tool, developed by the National Cancer Institute. **PA**: Pregnancy Physical Activity Questionnaire to calculate average weekly energy expenditure	Encouraged to track weight, food, and exercise regularly. Participants could use any scale available to them to measure weight, and any method they chose to track diet and activity (coaches suggested options such as the MyFitness- Pal and LoseIt website/phone apps, the CalorieKing Food and Exercise Journal, and the notes section of their phone)	Social Cognitive Theory and FRAMES model	**Acceptability**: Call attendance, average call length, use of the intervention website and online questionnaire completed at the end of the study
Phelan et al. (2023)	Combined lifestyle intervention (diet + PA)	**Intervention**: Standard care + education (2 × 20 min meetings with study team on preconception health + weight loss intervention (nutrition + PA + behavioural)	Ongoing, individual contact with a study interventionist. Visits conducted in person, on the phone, or through video conferencing. **First 16 weeks**: Weekly meeting for ~ 30 min	**First 16 weeks**: **Diet**: Prescribed a standard, low-fat diet (35% fat, 20% protein, 45% CHO). Calorie goals are based on baseline body weight with 300 kcal/day adjustments for breastfeeding status, if applicable. - Participants with an entry weight of < 91 kg are prescribed a 1200-kcal/day self-selected diet. - Participants with an entry weight of > 91 kg are prescribed a 1500-kcal/day. **PA**: Increase PA to at least 150 min per week (e.g., 30 min per day, 5 days per week)	**Measurement timepoints**: Baseline (preconception), 16 weeks and 26 weeks’ gestation. **Diet**: 24-h recalls on 2 random days over a week and completed in an interview format using the NCI Automated Self- Administered 24 h recall (ASA24). Assessing total Calories, kcal/d and % Calories Carbohydrates, Fats, Proteins	Weekly behavioural assignments reviewed by the interventionist. Given self-monitoring records or encouraged to use apps, if preferred	Social Cognitive Theory and “teachable moments” model	**Acceptability**: Attendance at intervention sessions and number of daily self-monitoring records returned
		**Aim**: To produce 10% WL over 16 weeks and WL maintenance until conception (1–3 years) **Control**: Standard care + education 2 × 20 min meetings with study team on preconception health	**After 16 weeks until conception**: bi-weekly meetings (or more frequently in the context of weight regain) to maintain weight loss until conception **After conception**: intervention contacts discontinued.	**After 16 weeks until conception**: **Diet**: Goals are adjusted to maintain WL or weight management goal until conception.**PA**: Advised higher goals (60 min/day). Brisk walking, “child-friendly,” and inexpensive activities, taking into consideration potentially unsafe neighbourhoods. Provided a pedometer and encouraged to gradually increase the number of steps they walk per day (with an increase of ~ 250 steps/day each week) until reaching 10,000 steps per day	**PA**: Wrist-worn actigraph accelerometer (MTI, Inc.) for 7 days. Assessing Total MVPA (unbouted), min/d and time watching TV/video programming (total h/wk). **Validated questionnaires**: The Weight Control Strategies Scale and The Eating Inventory			
Price et al. (2021)	Diet	**Intervention**: Very Low Energy Diet **Control**: Standard Hypocaloric Diet Intervention	**3 Phases**: **Phase 1 (Week 0–12)** Seen fortnightly. In-person. **Phase 2 (Week 13–16)**: Received 1-hour dietary counselling to assist weight maintenance at end of week 16	**Intervention**: **Phase 1 (Week 0–12): Very Low Energy Diet** - Replace two meals per day with Optifast ^®^ (Nestle Nutrition, Australia). - Third meal consisted of 150 g of protein (meat, chicken, fish, tofu, eggs), 2 cups of low-starch vegetables and salad dressed with 2 tablespoons of oil. - Group received ‘Allan Borushek’s Calorie, Fat and Carbohydrate Counter’ book. - Total daily calorie intake is approximately 800 cal. Over 12 weeks, 10–15% total body weight loss is expected	Data not reported	Access to a dietitian throughout study for dietary counselling	Data not reported	Data not reported
			**Phase 3a (Week 17–60)**: 30-minute study visits every 12 weeks until conception	**Phase 2 (Week 13–16): Weight Maintenance** - Adopt a healthy balanced diet based on the “Australian Guide to Healthy Eating” for a period of 4 weeks				
			**Phase 3b (Pregnancy)**: Medical and obstetric data collected throughout the pregnancy by attending midwife and/or obstetrician	**Phase 3a (Week 17–60)**: Ongoing weight maintenance whilst trying to get pregnant. **Phase 3b (Pregnancy)**: Standard pregnancy care with obstetric care provider.				
				**Control**: **Phase 1 (Week 0–12): Standard Hypocaloric Dietary Intervention** Nutritional advice delivered by a qualified dietitian in accordance with the ‘Australian Guide to Healthy Eating’. Daily energy expenditure calculated based on Basal Metabolic Rate and Activity Level (Harris-Benedict formula). A hypocaloric diet (500 kcal deficit from the daily energy expenditure) was recommended to achieve a weight loss of 0.5 kg per week. A food diary and an ‘Allan Borushek’s Calorie, Fat and Carbohydrate Counter’ book was provided to participants as tools to assist weight loss				
				**Phase 2 (Week 13–16): Weight Maintenance** Same as intervention phase 2.**Phase 3a (Week 17–60)**: Ongoing weight maintenance whilst trying to get pregnant				
				**Phase 3b (Pregnancy): Standard pregnancy care with obstetric care provider**				
Rönö et al. (2018)	Combined lifestyle intervention (diet + PA)	**Intervention: **Structured educational visits with the study nurse, in addition to standard antenatal clinic. Study visits included personalised dietary and physical activity counselling.	**Pre-pregnancy: **Participants visited the study nurse every 3 months	**Diet:** Encouraged to increase intake of vegetables, fruits, whole grains, low-fat dairy, vegetable fats high in unsaturated fats, fish, and lean meats, while reducing sugar-rich foods	**Diet**: 48-item food frequency questionnaire.The food groups included were low-fat milk (≤ 1% fat), low-fat cheese (≤ 17% fat), whole-grain cereals, fruits and berries, vegetables and legumes, fish, animal protein, sugar sweetened beverages, snacks, and fast food. Assessed quality of diet with dietary index (the maximum points being 17).			
		**Control**: Routine antenatal care + informational leaflets on healthy diet and exercise	**During pregnancy**: Participants had structured educational visits with the study nurse once per trimester, in addition to standard antenatal clinic visits. Study visits included personalised dietary and physical activity counselling	**PA**: Advised to engage in at least 150 min of moderate-intensity physical activity per week and adopt an active lifestyle. Study nurse created a personalised physical activity plan, updated throughout the study. Pedometers were provided as motivation	**PA**: Self-reported leisure time physical activity	Data not reported	Data not reported	Data not reported
Sujan et al. (2025)		**Intervention**: TRE and increased PA with two optional supervised exercise sessions week 2 and 4 in pre-prepregnancy period		**Diet**: Restricted daily time window of energy intake to ≤ 10 h, ending no later than 7 pm, for a minimum of five days per week throughout the study period. On the “off” days, the participants could choose their time window for energy intake. Apart from current recommendations about preconception/pregnancy nutrition, no advice about dietary composition or the amount of energy the participants should consume, and they could consume non-energy drinks outside the time window	**Diet**: Self- reported daily time window of energy intake for four days (three week and one weekend day) every eighth week in a printed study handbook.			
	Combined lifestyle intervention (diet + PA)	**Control**: Standard care and continued with habitual PA and dietary intake	Assessments were performed at baselines, week 8 in prepregnancy period, and at gestational weeks 12 and 28	**PA**: Instructed to obtain ≥ 100 weekly PAI points throughout the study period. PAI translates heart rate during physical activity into a score. Exercise training consisted of endurance exercise with the aim of high intensity. participants in the intervention group with a brochure suggesting various HIIT sessions to complete at home. Once pregnant, participants to choose between repeated short (30 s) work bouts at high intensity with low to moderate intensity periods in between or longer work periods with an intensity up to 85% of heart rate maximum	**PA**: Smartwatches (Amazfit GTS or Polar Ignite 2) captured PAI data	Reminders were sent to all participants by text message to complete dietary reporting and contacted the participants in the intervention group to offer additional supervised exercise sessions if they were not reaching the PAI target	Data not reported	Participants were categorised as adherent to time restricted eating if they reported a time window for energy intake of ≤ 10 h on at least two of these four days and adherent to exercise training if they earned and maintained ≥ 75 PAI points per rolling week

*CHO* Carbohydrates, *DASH*  Dietary Approaches to Stop Hypertension, *FRAME*  Feedback, Responsibility, Advice, Menu of options, Empathy, and Self-Efficacy, *HIIT*  High Intensity Interval Training, *Kcal*  kilocalorie, *PA*  physical activity, *PAI*  personal activity intelligence, *TRE*  time restricted eating, *WL*  weight loss

**Table 3:** Outcomes of studies

References	Intervention duration	GDM outcomes	Other reported outcomes	Changes in diet and/or PA outcomes	Feasibility and acceptability outcomes
Bogaerts et al. (2025)	Data not reported	**GDM occurrence** **Intervention** *n* = 19 (10.1%) **Control** *n* = 16 (8.3%) Unadjusted OR, 1.24; 95% CI, 0.62–2.50; *P* = 0.54 Adjusted OR* Adjusted for whether participant had GDM in a previous pregnancy (95% CI): 1.36 (0.64–2.86) *P* = 0.43	**Prepregnancy overweight or obesity** **Intervention** *n* = 93 (48.7%) **Control**: *n* = 95 (48.4%) Unadjusted OR, 1.01; 95% CI 0.68–1.50; *P* = 0.97	Data not reported	**Adherence** **Phase 1**: 94.8% attended at least one coaching session. 77.8% used the INTER-ACT application (median usage duration of 61 days (interquartile range, Q1 = 31, Q3 = 104 days))
			**Excessive GWG in subsequent pregnancy** **Intervention** *n* = 100 (51.8%) **Control** *n* = 117 (59.3%) Unadjusted OR, 0.74; 95% CI 0.49–1.10; *P* = 0.13		**Phase 2**: 55% attended at least one coaching session. 37.8%, used the INTER-ACT application (median usage duration of 12 days (interquartile range, Q1 = 3, Q3 = 44 days))
LeBlanc et al. (2021a, 2021b, 2022)	**Mean duration between baseline and conception**: 17.9 months	**GDM occurrence** **Intervention** *n* = 22 (24.7%	**Preconception weight loss** **Intervention** 3.7 (− 4.6–12.0) kg (3.5% of randomisation weight) **Control** 0.6 (− 7.5–8.7) kg (0.5% of randomisation weight) *P* < 0.001 **Change in BMI** **Intervention** − 1.32 (− 4.18–1.54) kg/m^2^ **Control** 0.25 (− 2.68–3.18) kg/m^2^ *P* = 0.02	**From Study Entry to mid-pregnancy**: Diet*: Fruit intake: **Intervention** + 0.67 cups **Control**: +0.06 cups *P* = 0.02 **PA**: **Vigorous-intensity activity**: **Intervention** 3.9 (− 1.6–9.4) Met-hr/week	**Acceptability**: **Completion rates (Average call duration)**: Weekly phone calls: 70.9% (21.8 min) Monthly phone calls: 73.3% (20.1 min) Intervention satisfaction: High (> 80%)
		**Control** *n* = 28 35.4% Unadjusted OR (95% CI): 0.60 (0.31–1.17) Adjusted OR* Adjusted for whether participant had GDM in a previous pregnancy (95% CI): 0.67 (0.33–1.36)	**Gestational Weight Gain** **Second Trimester** **Intervention** 0.42 (0.16–0.68) kg/wk **Control** 0.33 kg (0.05–0.61 kg) kg/wk *P* = 0.04 **Third Trimester** **Intervention** 0.56 (0.19–0.93) kg/week	**Control**: 1.2 (− 1.8 to 4.2) Met-hr/week *P* = 0.002 **Sports/ exercise engagement**: **Intervention** 17.0 (2.9–31.1) Met-hr/week **Control** 11.0 (1.5–20.5) Met-hr/week *P* = 0.03	
		**Early GDM screening** *n* = 116 (69% of cohort) **Intervention**: 13.6% **Control**: 29.8%	**Control** 0.43 (0.10–0.76) kg/week *P =* 0.02 **Total Gestational Weight Gain** **Intervention** 13.24 (5.04–21.44) kg **Control** 10.32 (2.91–17.73) kg *P* = 0.03	**Changes in sedentary time**: **Intervention** −4.9 (− 19.9–10.1) Met-hr/week **Control** 0.5 (− 7.1–8.1) Met-hr/week *P* = 0.02	
Phelan et al. (2023)	Mean duration between the baseline visit and conception: 49.8 (40.0) weeks after randomisation	**GDM occurrence** **Intervention** *n* = 22 (57.9%)	**Preconception Weight Loss**: **At 16 weeks** **Intervention**: *n* = 34 4.8 (3.5–6.0) kg (5.7% weight loss)	**Diet**: **Total Calories** **At Study Entry**: **Intervention**: *n* = 35 1,624 (1,458–1,772) kcal/d **Control**: *n* = 22 1,724 (1,515–1,933) kcal/d **Change from Study Entry to 16 weeks**: **Intervention**: *n* = 26 − 43.5 (− 327–240) kcal/d **Control**: *n* = 22 − 139.5 (− 389 to 111) kcal/d Between group differences: 93.7 (− 262 to 450); *P* = 0.60	**Acceptability**: Attendance rates at core measurement visits: 16 weeks: 88.9% 26 weeks’ gestation: 88% 6 weeks postpartum: 85.5% *P* > 0.05 between groups
	**Intervention** 53.0 (44.3) weeks **Control** 44.7 (32.1) weeks Between group difference: *P* = 0.36	**Control** *n* = 11 (44.0%) OR [95% CI], 1.84 (0.59–5.8))	**Control**: *n* = 22 0.7 (− 0.9–2.3) kg (0.7% weight loss) Between group difference: 4.1 (2.0–6.2) kg; *P* = 0.001	**Change from Study Entry to 26 weeks of gestation**: **Intervention**: *n* = 20 145.1 (− 168 to 459) kcal/d **Control**: *n* = 18 104.1 (− 164 to 372) kcal/d Between group differences: 55.3 (− 342 to 452); *P* = 0.78 **Weight control practices**,** total score** **At Study Entry**: **Intervention**: *n* = 38.6 (59.0–69.3) **Control**: *n* = 24: 63.4 (56.7–69.9)	
				**Change from Study Entry to 16 weeks**: **Intervention**: *n* = 29 32.9 (25.4–40.3) **Control**: *n* = 23 9.0 (− 0.1 to 18.1) Between group differences: 24.9 (13.5–36.4); *P* < 0.0001 **Change from Study Entry to 26 weeks of gestation**: **Intervention**: *n* = 30 11.0 (3.7–18.3) **Control**: *n* = 21 5.7 (− 3.7 to 15.0) Between group differences: 6.0 (− 5.2 to 17.1); *P* = 0.29 **Dietary Restraint** **At Study Entry** **Intervention**: *n* = 38 11.1 (9.8–12.3) **Control**: *n* = 24 11.2 (9.7–12.7)	
				**Change from Study Entry to 16 week**: **Intervention**: *n* = 29 5.3 (3.4–7.2) **Control**: *n* = 23 0.7 (− 1.1 to 2.5) Between group differences: 5.3 (3.4–7.2); *P* = 0.001 **Change from Study Entry to 26 week of gestation**: **Intervention**: *n* = 30 3.1 (1.20–5.0) **Control**: *n* = 21 1.2 (− 0.7 to 3.1) Between group differences: 2.1 (− 0.5 to 4.8); *P* = 0.11 **PA**: **Total MVPA**	
			**At the final preconception visit (49.7 [SD**,** 39.9] weeks after randomization)**: **Intervention**: *n* = 35 4.1 (1.9–6.2) kg **Control**: *n* = 23 1.3 (− 1.4–4.0) kg Between group differences: 2.8 (− 0.7 to 6.3) kg; *P* = 0.12	**At Study Entry**: **Intervention**: *n* = 32 64.2 (60.1–68.3) min/day **Control**: *n* = 22 62.9 (57.9–67.9) min/day **Change from Study Entry to 16 weeks**: **Intervention**: *n* = 25) 15.1 (7.2–23.2) min/day **Control**: *n* = 20 5.5 (− 0.8 to 11.9) min/day Between group differences: − 9.9 (− 18.2 to 1.6); *P* = 0.02	
			**Achieved ≥ 5% weight loss by the final preconception visit** **Intervention**: *n* = 15 (42.9%) **Control**: *n* = 4 (17.4%) OR [95% CI], 6.9 (1.4–33.8) **Gestational Weight Gain**: **Intervention**: *n* = 30 12.7 (10.1–15.4) kg **Control**: *n* = 19 9.7 (6.3–13.1) kg *P =* 0.17 **Blood Glucose (mg/dL)**: **Study Entry**: **Intervention**: 84.9 (80.7–88.9) **Control**: 86.3 (81.2–91.3)	**Change from Study Entry to 26 weeks of gestation**: **Intervention**: *n* = 18 − 14.5 (5.59–23.0) min/day **Control**: *n* = 17 − 9.6 (3.1–16.1) min/day Between group differences: 4.8 (0.5–9.0); *P* = 0.03 **Time watching TV/video programming** **At Study Entry** **Intervention**: *n* = 38): 13.6 (11.1–16.0) hour/week **Control**: *n* = 23 13.9 (10.7–17.0) hour/week	
			**Change from Study Entry to 16 weeks**: **Intervention**: − 2.5 (− 2.9 to 8.0) mg/dL **Control**: − 5.2 (− 11.6 to 1.1) mg/dL Between-group differences: 3.0 (− 2.0 to 7.7); *P* = 0.446	**Change from Study Entry to 16 weeks**: **Intervention**: *n* = 29 − 3.1 (− 6.9–0.8) hour/week **Control**: *n* = 23 − 1.7 (− 5.8 to 2.5) hour/week Between group differences: − 1.3 (− 6.5 to 3.9); *P* = 0.61	
			**Change from Study Entry to 26 weeks of gestation** **Intervention**: − 4.9 (− 11.1 to 1.4) **Control**: − 13.8 (20.6 to − 7.0) Between-group differences: 9.1 (− 1.0 to 19.2); *P* = 0.08	**Change from Study Entry to 26 weeks of gestation**: **Intervention**: *n* = 28 − 1.0 (− 4.8 to 2.9) hour/week **Control**: *n* = 21 − 0.8 (− 5.1 to 3.5) hour/week Between group differences: − 0.6 (− 7.0 to 5.8); *P* = 0.85	
Price et al. (2021)		**GDM occurrence** **Intervention** *n* = 8 (23%) **Control** *n* = 10 (45%) OR (95% CI) 0.36 (0.1–1.31); *P* = 0.09	**Preconception Weight Loss** **Intervention** 13.0 kg (completer-only analysis (13.0 kg) = 95% CI 12.4–13.6; ITT analysis (11.2 kg) = 95% CI 9.4–13.1) **Control** 3.2 kg (completer-only analysis (3.2 kg) = 95% CI 2.5–3.9; ITT analysis (2.1 kg) = 95% CI: 1.1–3.1) Between group difference: 9.8 kg (completer-only analysis (9.8 kg) = 95% CI 8.8–10.7; ITT analysis (9.2 kg) = 95% CI 7.6–10.8)	Data not reported	Data not reported
		**Fasting glucose (mmol/L) Intervention**: 7.6 (0.1) mmol/L	**Weight change prior to pregnancy**: (defined as the period between the end of the 12-week intervention and either pregnancy or week 60 if no pregnancy occurred)		
		**Control**: 4.8 (0.2) mmol/L *P* = 0.42	**Intervention** 3.6 (2.7–4.5) kg (+ 3.2% from post-intervention weight) **Control** 3.0 (2.3–3.7) kg (+ 2.9% from post-intervention weight) *P* = 0.84 **Gestational Weight Gain** **Intervention** 10.3 (9.3–11.3) kg		
			**Control** 10.9 (9.9–11.9) kg *P* = 0.66 **Fasting maternal plasma glucose at 26 to 28 weeks’ gestation** **Intervention** 4.6 (4.5–4.7) mmol/L **Control** 4.8 (4.6–5.0) mmol/L *P* = 0.42		
Rono et al. (2018), Valkama et al. (2018)	**Mean duration between baseline and conception** **Intervention** 4.6 (3.6) months	**GDM occurrence** **Intervention** *n* = 39 (60%) **Control** *n* = 34 (54%) *P* = 0.61	**Total Gestational Weight Gain** **Intervention** 9.6 (7.8–11.5) kg	**Diet**: **Dietary Index** **At Study Entry** **Intervention** 10.1 (7.3–12.9) points **Control** 10.4 (7.9–12.9) points	Data not reported
	**Control** 3.8 (3.7) months Between group difference: *P* = 0.26	**Early GDM diagnosis** (before 20 weeks, mean 13.3 ± 2.5 weeks)	**Control** 9.2 (7.6–10.8) kg *P* = 0.93	**From Study Entry to First Trimester** **Intervention** + 1.2 (0.4–2.1) points **Control** + 0.6 (− 0.1 to 1.4) points *P* = 0.28 **PA**: **LTPA**	
		**Intervention**: 64% **Control**: 56% *P* = 0.47 **GDM reoccurrence** **Intervention** *n* = 35 (90%) **Control** *n* = 31 (91%)	**Change in Fasting Plasma Glucose from Baseline to Third Trimester**: **Intervention** − 0.46 mmol/L **Control** − 0.61 mmol/L *P* = 0.77	**At Study Entry** **Intervention** 101 (26–176) min/week **Control** 100 (6–194) min/week **From Study Entry to First Trimester** **Intervention** + 24 (− 15 to 63) min/week **Control** + 12 (− 50 to 25) min/week *P =* 0.18	
Sujan et al. (2025)**	**Average time to pregnancy** **Intervention** 112 (105) days	**Two-hour plasma glucose concentrations at gestational week 28** **Intervention** *n* = 49 6.7 (1.5) mmol/L	Weight*** **At Baseline** **Intervention** 81.1 (16.0) kg **Control** 81.5 (13.2) kg **Prepregnancy week 8** **Intervention** 81.6 (12.5) kg	**Self-reported total energy intake** Between group differences (Intervention – control) Before pregnancy: −57.0 kcal/day, (− 183.9 to 69.9, *P* = 0.38)	Data not reported
	**Control** 84 (69) days *P* = 0.10	**Control: n = 53** 6.4 (1.1) mmol/L Mean difference 0.48 mmol/L, 95% confidence interval − 0.05 to 1.01, *P =* 0.08. **GDM occurrence**	**Control** 78.9 (14.8) kg Between group differences: − 0.9 (− 2.0 to 0.3); *P* = 0.15 **Gestational week 12** **Intervention** 78.8 (15.2) kg **Control** 79.5 (12.9) kg Between group differences: − 0.9 (− 2.2 to 0.3); *P* = 0.15	During first trimester: 41.2 kcal/day, −92.6 to 175.1, *P* = 0.55	
		**At week 12** **Intervention** *n* = 3 (5.9%) **Control** *n* = 3 (5.9%) *P =* 1.00 **At Week 28** **Intervention** *n* = 8 (16.3%) **Control** *n* = 6 (11.5%) *P =* 0.57	**Gestational week 28** **Intervention** 85.1 (12.3) kg **Control** 86.8 (12.3) kg Between group differences: − 2.0 (− 3.3 to − 0.8); *P* = 0.002	During second trimester: 39.7 kcal/day, − 101.4 to 180.9, *P* = 0.58 During third trimester: − 110.3 kcal/day, − 248.0 to 27.3, *P* = 0.12	

*CI* Confidence Interval, *Kg* kilogram, *LTPA* leisure time physical activity, *Mmol/L* millimole per litre, *MVPA* moderate-vigorous physical activity, *PA* physical activity

*LeBlanc et al. (2021a, 2021b) reported no significant differences on other dietary intake patterns

**All results reported for Sujan et al. (2025) reflect intention-to-treat analyses. The results from the per protocol analyses were similar to those from (except for time to pregnancy which was significantly longer in the intervention group (48 days, 95% confidence interval 15 to 81, *P* = 0.005)

***Sujan et al. (2025) reported no little or no evidence of other between group differences during pregnancy

In relation to behaviour change outcomes, changes in moderate-vigorous PA levels from study entry to 16 weeks increased in the intervention versus control group (*P* = 0.02). This pattern was also observed up to 26 weeks’ gestation *(P =* 0.03). No further effects on changes related to dietary intake or PA were reported.

Price et al. (2021) conducted a multiple site, parallel-group RCT which aimed to determine if nonsurgical, substantial weight loss can be achieved in women with obesity prior to pregnancy, using a modified very low energy diet (VLED) programme. Additionally, they sought to determine whether weight loss improved glucose metabolism and obesity-related adverse pregnancy outcomes for both the woman and her offspring (Price et al., 2021). The trial recruited women with a BMI of 30–55 kg/m^2^ who were planning a pregnancy in the next 6 to 12 months. Both groups (intervention, *n* = 85; control, *n* = 79) took part in a 12-week diet-only intervention, followed by a 4-week weight maintenance period prior to trying to conceive. The intervention arm required participants to follow a VLED, replacing two meals per day with a commercially available VLED formulation, Optifast^®^ (Nestle Nutrition, Australia). In contrast, the control participants followed a standard hypocaloric diet (500 kcal deficit from their daily energy expenditure) with advice delivered by a qualified registered dietitian (Table 2). Weight loss at the end of the 12-week intervention was different between groups (intervention, 11.9% (13.0 ± 0.5 kg); control, 3.1% (3.2 ± 0.6 kg); *P* < 0.01), respectively. Between the start of the intervention and either conception or week 60 of the study if conception did not occur, weight regain did not differ between groups (intervention, 3.2% (3.6 ± 0.9 kg); control, 2.9% (3.0 ± 0.7 kg); *P* = 0.84). In a per protocol analysis (intervention, *n* = 50; control, *n* = 32), the median time to pregnancy was significantly different between groups (intervention, 51.0 days (32.0 d, 169.0 d); control, 140.5 days (75.3 d, 211.0 d); *P* = 0.03). No difference in the primary outcome of fasting maternal plasma glucose at OGTT of 26 to 28 weeks’ gestation between groups was observed. Similarly, post hoc analysis of the 1-hour post glucose load demonstrated no difference between groups. However, 2-h post glucose load was higher in participants randomised to the intervention group compared with the control group (5.9 (0.2); 6.7 (0.3) mmol/L, respectively; *P* = 0.04) (Table 3).

### Pre-pregnancy and Pregnancy Interventions

Four interventions were delivered prior to conception and continued throughout pregnancy (Bogaerts et al., 2025; LeBlanc et al., 2021b; Rönö et al., 2018; Sujan et al., 2025). All trials were combined interventions, focused on modifying both dietary intake and PA, with Bogaerts et al. (2025) also including mental health wellbeing (Table 2).

The Interpregnancy and pregnancy lifestyle intervention (INTER-ACT) was a multi-centre RCT, which aimed to assess the effectiveness of behaviour change intervention that began interconception and continued through the subsequent pregnancy in women with a history of excessive GWG (Bogaerts et al., 2025). The trial recruited women within 6 weeks of delivery whose GWG exceeded National Academy Medicine recommendations (Rasmussen & Yaktine, 2009). During the interpregnancy period, the intervention group (*n* = 193) had access to 4 face-to-face coaching sessions and an e-health application which promoted healthy behaviours, such as improving dietary quality, supporting the return to pre-pregnancy weight, engaging in PA and addressing mental health wellbeing. Upon a subsequent pregnancy, the intervention group then had 3 face-to-face coaching sessions and continued access to the app. The control group (*n* = 197) received home visits at 6 weeks and 6 months postpartum, and subsequently every 6 months until the start of their next pregnancy and standard antenatal care during pregnancy. There was no significant difference in the primary outcome of composite pregnancy and birth related outcomes or solely GDM (Odds Ratio (OR), 1.24; 95% Confidence Interval (CI), 0.62–2.50, *P* = 0.054) between groups, Table 3).

The PREPARE trial was a single-centre RCT, which aimed to determine whether pre-pregnancy weight loss reduced GWG and improved pregnancy outcomes (LeBlanc et al., 2021b). The trial recruited women with a BMI of ≥ 27 kg/m^2^ who were planning pregnancy within the next 2 years. During pregnancy, both groups (intervention, *n* = 164; control, *n* = 162) received standard antenatal care through their obstetrical provider and information on having a healthy pregnancy. The intervention group also received individualised diet and PA advice during weekly health coach and behavioural interventionist-led counselling sessions for the first six months, then monthly for the following 18 months or until the end of pregnancy. A total of 33.1% and 50.9% of women conceived within 6 months and 6–24 months of study commencement, respectively, with no significant difference between groups (*P >* 0.05*)*. There was a significant difference in preconception weight loss (intervention, 3.7 (8.3) kg (3.5% of randomisation weight); control, 0.6 (8.1) kg (0.5% of randomisation weight); *P* < 0.001) and a reduction in BMI (intervention, 1.32 (2.86) kg/m^2^; control, 0.25 (2.93) kg/m^2^; *P* = 0.02) between groups. Mean GWG was higher in the intervention group in the second and third trimesters, leading to higher overall weight gain among the intervention group (intervention, 13.24 (8.20) kg; control, 10.32 (7.41) kg; *P* = 0.03). There was no effect of the intervention on the secondary outcome of GDM. In a secondary analysis of the trial, 59 (66%) intervention participants and 57 (72%) control participants received randomised early OGTT screening (LeBlanc et al., 2021a). Of the intervention group who received early screening, 13.6% were diagnosed with GDM, compared to 29.8% of those in the control group. Furthermore, those in the intervention group were 73% less likely to be diagnosed with GDM in early pregnancy compared to those in the control group (OR, 0.27; 95% CI, 0.09–0.80).

Some changes in PA and diet were reported in the PREPARE trial (LeBlanc et al., 2021b) (Table 3). In an additional secondary analysis (LeBlanc et al., 2022), from randomisation to mid-pregnancy, vigorous‐intensity activity (intervention, 3.9 (5.5) Met‐hr/week; control, 1.2 (3.0) Met‐hr/week; *P* = 0.002) and sports/exercise (intervention, 17.0 (14.1); control, 11.0 (9.5) Met‐hr/week; *P* = 0.03) were greater in the intervention group. The intervention and control groups also differed in changes in sedentary time (intervention, − 4.9 (15.0); control, + 0.5 (7.6) Met‐hr/week; *P* = 0.02). Additionally, participants in the intervention group had larger increases in fruit intake than those in the control group (intervention, + 0.67; control, + 0.06 cups; *P* = 0.02).

The RADIEL trial was a multi-centre RCT, which aimed to evaluate the efficacy of a combined diet and PA pregnancy intervention on GDM prevention (Koivusalo et al., 2016). A secondary publication included in this review (Rönö et al., 2018) focused on combined diet and PA intervention during the pre-pregnancy period. The trial recruited women with a BMI ≥ 30 kg/m^2^ and/or women with previous GDM who were planning pregnancy within the following 12 months. Both groups (intervention, *n* = 116; control, *n* = 112) received standard antenatal care provided to all Finnish pregnant women by public primary healthcare centres and information on having a healthy pregnancy. This consisted of 10–15 visits to a nurse and 2–3 visits to a physician during pregnancy and information leaflets on healthy diet and exercise. In addition, the intervention group also received nurse-led personalised dietary and PA counselling every three months during pre-pregnancy and once per trimester during pregnancy (Table 2). Within 12 months from study commencement, 62.5% of women conceived, with no significant difference between intervention and control groups (*P* = 0.84). There was no effect of the intervention on the primary outcome of GDM. In a secondary analysis of the dietary intake during the RADIEL trial, found that pre-pregnancy lifestyle counselling had limited effect on improving diet, with only the intake of low-fat cheese increasing in women who did not develop GDM (*P* = 0.028) (Valkama et al., 2018) (Table 3).

The BEFORE THE BEGINNING trial was a single-centre RCT, which aimed to determine the effect of a pre-pregnancy behaviour change intervention on glucose tolerance in women at higher risk of developing GDM (Sujan et al., 2025). The trial recruited women contemplating pregnancy within the next 6 months and with at least one of the following criteria: BMI ≥ 25 < 40 kg/m^2^, GDM in a previous pregnancy, fasting plasma glucose > 5.3 mmol/L, previous newborn > 4.5 kg, or Non-European ethnicity. The intervention group (*n* = 83) followed time restricted eating (TRE), which restricted the daily time window of energy intake to ≤ 10 h, ending no later than 7 pm, for a minimum of five days per week throughout the study period, and exercise training during the pre-pregnancy and pregnancy periods (Table 2). The control group (*n* = 83) received standard prenatal and antenatal care and advice and were encouraged to continue habitual PA and dietary intake. There was no difference in the primary outcome of two-hour plasma glucose concentrations at gestational week 28 (Table 3). Similarly, there was no difference in GDM diagnosis at gestational week 12 or gestational week 28 between groups (*P* > 0.05*)*. Preconception weight loss did not differ between groups, however GWG was different; weight gain in the intervention group at gestational week 28 was 2 kg lower (95% CI, − 3.3 to − 0.8, *P* = 0.002), and fat mass gain was 1.5 kg lower (− 2.5 to − 0.4, *P* = 0.008) than the control group.

### Risk of Bias

**Fig. 2:**
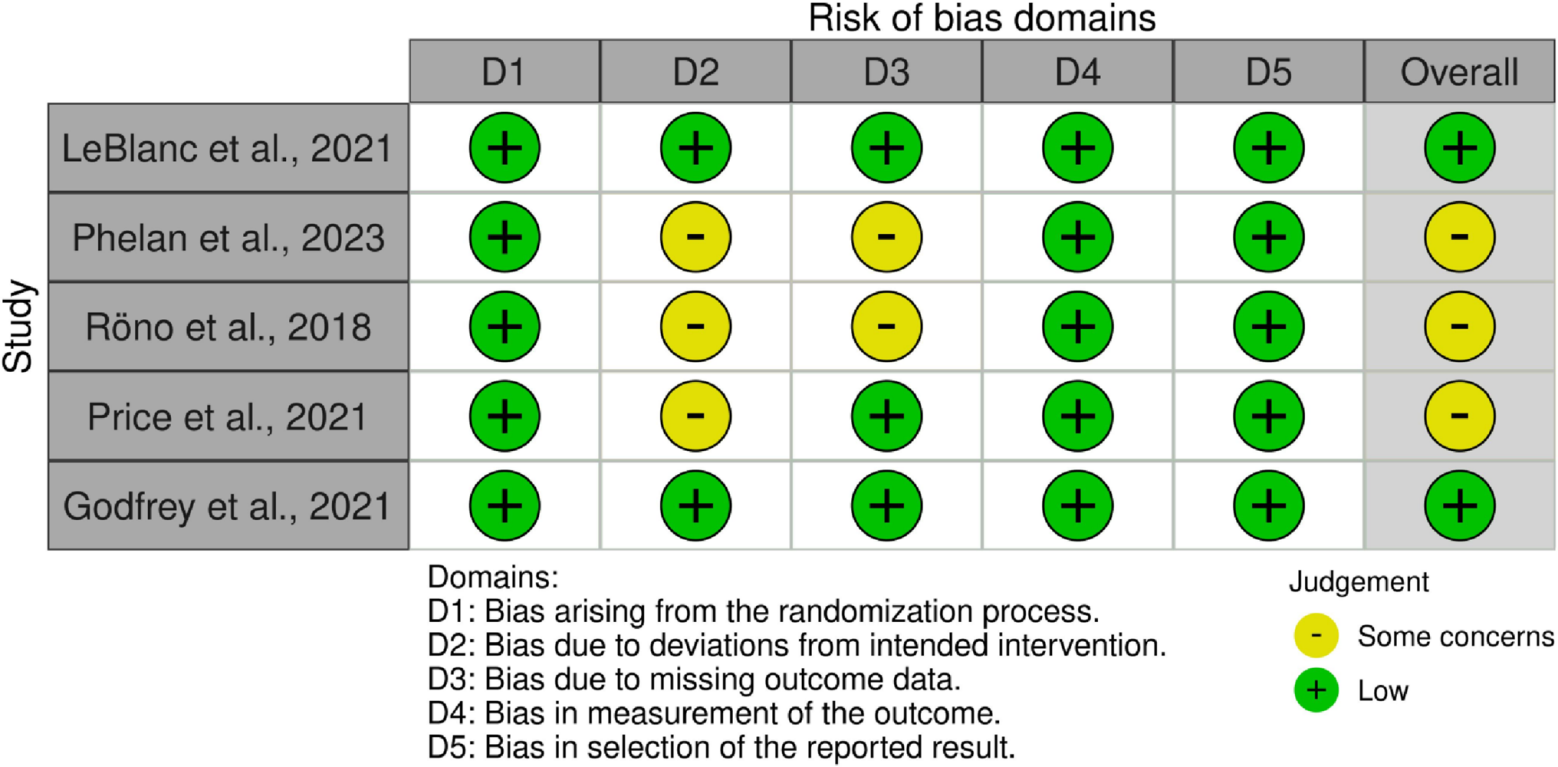
Risk of bias assessment of intervention studies using Cochrane RoB 2-tool

The overall quality of the included studies varied and is summarised in Fig. 2. One study was assessed as ‘low risk of bias’ (LeBlanc et al., 2021b), and five were assessed as ‘some concerns’ (Phelan et al., 2023; Price et al., 2021; Rönö et al., 2018). The deviations from the intended intervention and missing outcome data metrics were adequate for five studies. Five trials were underpowered at analysis, with three reporting that the conception rate was lower than expected (Phelan et al., 2023; Price et al., 2021; Rönö et al., 2018).

## Discussion

This systematic review aimed to evaluate preconception interventions in women at high risk of developing GDM. The findings of the review highlight the paucity of interventions which aim to optimise pre-pregnancy health and reduce the risk of developing GDM in higher risk women. Six RCTs equating to nine publications were identified, with intervention components focusing on modification of dietary intake and/or increase in PA. Although some studies reported improvements in diet and PA, and differences in preconception weight loss and/or GWG, no studies reported a reduction in GDM. The results were limited by small sample sizes, and five of the six trials were underpowered to detect a difference in GDM. GDM as a primary or secondary outcome was observed across the six trials; and additionally, none of the included studies published after 2020 formally utilised the core outcome set (COS) published for GDM prevention and treatment (Egan et al., 2020).

### Effectiveness of Preconception Interventions in GDM Prevention

A previous systematic review investigating the effects of diet and PA during the preconception period, found no significant difference in rates of GDM (Poprzeczny et al., 2025). The current review focused specifically on women at high risk of developing the condition and extended the scope beyond diet and physical activity interventions to include additional types (nutritional supplementation and pharmacological interventions), providing further evidence to higher-risk preconception populations. Half of included trials aimed to reduce weight before pregnancy in women living with overweight or obesity. These trials reported weight loss in the intervention groups compared to the control groups using either behaviour change advice (related to diet, PA) or a VLED. The interventions reduced weight before pregnancy and improved dietary and PA behaviours, but there was no reduction in GDM. These relatively small trials were underpowered to detect effects of the impact of preconception weight loss on GDM, highlighting the need for further research in this area.

BEFORE THE BEGINNING trial reported lower GWG in the intervention group, while the PREPARE trial reported higher GWG, and Price et al. (2021) observed no differences between groups. Given that none of these trials, nor Phelan et al. (2023) reported a reduction in GDM in women with a high BMI, there is a need to re-evaluate assumptions about intervention targets. It is important to note that there was a heterogeneity across BMI cut-offs used throughout the included studies (ranging from ≥ 25 kg/m^2^ to between 30 and 55 kg/m^2^). While the GWG observed in the intervention arms may have negated any effect of intervention on GDM rates, evidence suggests that maternal BMI and GWG likely represent independent pathways to adverse antenatal outcomes rather than BMI acting through GWG, as was traditionally assumed. This decoupling of pathways may explain why GWG-focused interventions have failed to reduce GDM across trials (Dodd et al., 2024b). Interventions may therefore need to target these specific mechanisms and/or be tailored to phenotype and timing, distinguishing early-identified GDM (< 20 weeks) from later-onset GDM (≥ 20 weeks) (Simmons et al., 2024). Overall, greater public health awareness, larger-scale interventions, and further research are needed to address overweight and obesity in women of reproductive potential and to develop effective obesity management strategies before pregnancy.

Beyond behaviour change intervention, the evidence supporting the use of myo-inositol supplementation during pregnancy to prevent GDM is growing (Mashayekh-Amiri et al., 2022). The NiPPeR trial aimed to investigate the effect of myo-inositol, probiotic, and micronutrient nutritional supplementation before pregnancy on maternal glycemia and pregnancy outcomes (Godfrey et al., 2021). The intervention did not reduce GDM, and further subgroup analysis of higher-risk women (i.e. women with overweight or obesity or those with documented dysglycaemia) did not show any benefit however it was not powered to do so. Further research on the use of supplementation such as myo-inositol in reducing GDM in women with risk factors before pregnancy in adequate powered studies is warranted.

### Behavioural Changes

Evidence suggests that antenatal diet and PA interventions have modest effects in preventing GDM in high-risk populations (Quotah et al., 2024). Five of the six RCTs (Bogaerts et al., 2025; LeBlanc et al., 2021b; Phelan et al., 2023; Rönö et al., 2018; Sujan et al., 2025) included in this review utilised combined behaviour change interventions focused on modifying both dietary intake and PA and facilitating behaviour change education. While no study reported reducing GDM, improved dietary behaviours were reported such as increases in fruit, vegetable, and low-fat dairy intake. Three of the interventions (Bogaerts et al., 2025; LeBlanc et al., 2021b; Phelan et al., 2023) alluded to behaviour change underpinnings of Social Cognitive Theory (LeBlanc et al., 2016; Phelan et al., 2021), ‘teachable moments’ (Phelan et al., 2021), the FRAMES model (LeBlanc et al., 2016), motivational interviewing and behaviour change techniques (Bogaerts et al., 2017) in their protocols, however, the trials did not discuss their chosen behaviour change theories relative to their findings. Promoting behaviour change to improve health outcomes through applying established theories and frameworks is important and should be considered in future intervention design (Suto et al., 2025). However, the needs of women between pregnancies may differ considerably from those experiencing pregnancy for the first time and should also be considered. Observational studies suggest that women with a previous pregnancy are often less prepared for a subsequent pregnancy in relation to health behaviours and have additional challenges such as childcare (McLeish et al., 2020; Slomian et al., 2021). As such, future preconception interventions should be suitably tailored to consider individual needs of women.

### Feasibility and Acceptability of Interventions

The studies included in this review provide evidence into recruitment and retention when undertaking intervention studies during the preconception and interconception periods. High attrition rates, compounded by participant dropout and participants not experiencing a pregnancy, led to five studies being underpowered. Feasibility and acceptability were assessed through session attendance, self-monitoring, feedback and intervention engagement. Phelan et al. (2023) reported high early engagement which reduced as the intervention progressed, which highlights the need for ongoing participant engagement strategies, tailored to intervention stage. PREPARE (LeBlanc et al., 2021b, 2022) and RADIEL (Rönö et al., 2018) both highlighted the role of personalised counselling and digital tools in sustaining engagement, but logistical challenges, including scheduling and participant workload, were reported. Price et al. (2021) found in-person counselling effective for dietary adherence but resource-intensive. Meanwhile, INTER-ACT found that coaching session attendance and app engagement were higher during interpregnancy than during the subsequent pregnancy (Bogaerts et al., 2025). The use of digital intervention show promise for improving diet, PA (Farzandipour et al., 2020) and postpartum diabetes prevention (O’Reilly et & Laws, 2019), suggesting blended, or digital approaches may support GDM prevention efforts in the preconception period (O’Connor et al., 2024), but may need additional consideration during pregnancy.

### Ongoing RCT Intervention Studies

Additional published protocols of RCTs (Chan et al., 2022; Kornerup et al., 2025; Mitchell et al., 2025) on preconception interventions exploring strategies to improve health outcomes in women with high risk are ongoing (Table 4). The ‘Healthy Early Life Moments in Singapore’ (HELMS) study is a single-arm implementation study which aims to examine whether an integrated behaviour change intervention initiated at preconception and continuing throughout pregnancy and postpartum can improve metabolic and mental health of women living with overweight and obesity and improve early child growth (Chan et al., 2022). The primary outcome is pregnancy rate, and co-primary outcomes include metabolic health, including GDM diagnosis, and mental health status. The Begin Better trial in Australia is a parallel RCT which aims to conduct a behaviour change intervention for women with overweight or obesity who are planning pregnancy and evaluate the impact of this intervention on maternal health and well-being prior to conception, and on pregnancy, birth and newborn health outcomes in a subsequent pregnancy. The primary outcome is infant birth weight z-score, with secondary maternal and infant outcomes, including GDM (Mitchell et al., 2025). The PRE-STORK trial in Denmark is a single-centre, parallel, RCT that will investigate the effect of a behaviour change intervention before and during pregnancy in women with overweight or obesity on neonatal adiposity measured at birth. The primary outcome is the difference in neonatal adiposity between groups. GDM diagnosis is a secondary outcome (Kornerup et al., 2025).

**Table 4:** Protocols of on-going RCT intervention studies

Title	Country	Study design	Study aim
Effects of an integrated mobile health lifestyle intervention among overweight and obese women planning for pregnancy in Singapore: protocol for the single-arm healthy early life moments in Singapore (HELMS) study	Singapore	Single-arm implementation study	To examine whether an integrated lifestyle intervention initiated at preconception and continuing throughout pregnancy and postpartum periods can improve the metabolic and mental health of women with overweight and obese and improve early child growth.
Pre-conception lifestyle intervention to optimise maternal health for a better start to life: the BEGIN BETTER prospective randomised controlled trial protocol	Australia	Prospective, parallel-group, randomised controlled trial	To conduct a randomised controlled trial of a comprehensive lifestyle intervention prior to conception, for women with overweight or obesity who are planning pregnancy within 2 years and evaluate the impact of this intervention prior to conception on maternal health and well-being prior to pregnancy; and pregnancy, birth and newborn health outcomes in a subsequent pregnancy.
Healthy lifestyle before and during pregnancy to prevent childhood obesity: study protocol for a parallel group randomised trial - the PRE-STORK trial	Denmark	Single-centre, parallel-group, randomised controlled trial	To investigate the effect of a lifestyle intervention *before* and *during* pregnancy in women with overweight or obesity on neonatal adiposity measured at birth in their children.

These studies commenced in September 2020, February 2021 and September 2022 respectively, and peer-reviewed results are eagerly awaited.

### Strengths and Limitations

The present systematic review utilised a comprehensive search strategy and well-defined eligibility criteria to identify behaviour change, supplementation and/or pharmacological interventions in the pre-pregnancy period. The focus on RCTs offered an opportunity for a robust assessment of the cause-and-effect relationships between pre-pregnancy intervention and GDM prevention. The screening, data extraction and risk of bias assessments were performed in duplicate. However, the interpretation of the review is limited by the size, heterogeneity and quality of the studies included. In addition, five of the six RCTs were inadequately powered to determine the effect of pre-pregnancy intervention on preventing GDM. Furthermore, the review focused on individual-level factors that impact GDM risk, however, it should be noted that social and structural factors also play a role (Simmons et al., 2024).

Potential publication bias for some of the intervention effects was found, and therefore, the interpretation of the findings is limited by the possible bias from selective reporting. All included studies were conducted in high-income countries and in predominantly white ethnic populations, limiting generalisability to populations who have a higher risk of GDM. Additionally, the exclusion of non-English studies may have contributed to publication and selection biases.

### Recommendations for Further Research and Practice

Given the large proportion of women with risk factors and suboptimal health behaviours at the start of pregnancy and the limited timeframe to modify behaviours during pregnancy, there is a need for adequately powered preconception RCTs to reduce adverse pregnancy outcomes, including GDMThese studies should evaluate the effects of behavioural, supplementation, and pharmacological interventions, particularly in populations with specific ethnic and socioeconomic backgrounds where the need is greatest (Vounzoulaki et al., 2024). Technology may facilitate engagement and behaviour change strategies (Halligan et al., 2021; Lim et al., 2019). To date, preconception interventions have focused on either dietary and/or physical activity behaviour change or supplementation. The introduction of glucagon-like peptide-1 receptor agonists (GLP-1 RAs) into clinical practice warrants further exploration of pharmacological intervention to treat the disease of obesity before conception (Price & Nankervis, 2025). In a cohort study, prescription of GLP-1 RAs within 24 months preceding a pregnancy was associated with a reduced risk of several adverse obstetrical outcomes, including GDM (Imbroane et al., 2025), however, evidence from RCTs is warranted. Furthermore, COS related to GDM prevention and treatment (Egan et al., 2020) have been published, and relevant work related to wider preconception health is ongoing (Schoenaker et al., 2024). Future studies should consider the implementation of COS in their study design and implementation.

Finally, future studies should incorporate behaviour change theory in the design and implementation of interventions (Scott et al., 2022). Such interventions should also be co-designed with women and relevant stakeholders to determine effective recruitment and engagement strategies to optimise health before pregnancy to reduce GDM risk and support long-term prevention of T2D. This co-design process should also address societal GDM and weight-stigmatising attitudes and norms, alongside providing professional development for healthcare professionals. Both are important to eliminate stigma in the preconception period, which in turn can positively impact maternal and child health outcomes during pregnancy and beyond (Davidsen et al., 2022; Hill et al., 2023). Additionally, interventions must account for the mental health and psychosocial dimensions of GDM management, which play a key role in shaping how women engage with interventions (Faal Siahkal et al., 2022).

## Conclusion

The preconception period provides an opportunity to target modifiable risk factors in women most likely to develop GDM. This review demonstrates the paucity of studies that initiate intervention in the preconception period in higher risk women. The limited findings showed that while behaviour change interventions lead to improvements in diet, PA and differences in preconception weight and/or GWG, they did not reduce incidence of GDM. However, five out of the six RCTs included were underpowered. Adequately powered RCTs are needed which target modifiable risk factors prior to pregnancy in women who are more likely to develop GDM to improve immediate and long-term health of both women and their infants.

## Electronic Supplementary Material

Below is the link to the electronic supplementary material.

Supplementary Material 1

## Data Availability

Not applicable.
